# Painting of Fourth and the X-Linked 1.688 Satellite in *D. melanogaster* Is Involved in Chromosome-Wide Gene Regulation

**DOI:** 10.3390/cells9020323

**Published:** 2020-01-30

**Authors:** Samaneh Ekhteraei-Tousi, Jacob Lewerentz, Jan Larsson

**Affiliations:** Department of Molecular Biology, Umeå University, SE-90187 Umeå, Sweden; samaneh.tousi@umu.se (S.E.-T.); jacob.lewerentz@umu.se (J.L.)

**Keywords:** painting of fourth, dosage compensation, satellite DNA, heterochromatin, epigenetics, *Drosophila melanogaster*

## Abstract

Chromosome-specific regulatory mechanisms provide a model to understand the coordinated regulation of genes on entire chromosomes or on larger genomic regions. In fruit flies, two chromosome-wide systems have been characterized: The male-specific lethal (MSL) complex, which mediates dosage compensation and primarily acts on the male X-chromosome, and Painting of fourth (POF), which governs chromosome-specific regulation of genes located on the 4th chromosome. How targeting of one specific chromosome evolves is still not understood; but repeated sequences, in forms of satellites and transposable elements, are thought to facilitate the evolution of chromosome-specific targeting. The highly repetitive 1.688 satellite has been functionally connected to both these systems. Considering the rapid evolution and the necessarily constant adaptation of regulatory mechanisms, such as dosage compensation, we hypothesised that POF and/or 1.688 may still show traces of dosage-compensation functions. Here, we test this hypothesis by transcriptome analysis. We show that loss of *Pof* decreases not only chromosome 4 expression but also reduces the X-chromosome expression in males. The 1.688 repeat deletion, *Zhr^1^ (Zygotic hybrid rescue*), does not affect male dosage compensation detectably; however, *Zhr^1^* in females causes a stimulatory effect on X-linked genes with a strong binding affinity to the MSL complex (genes close to high-affinity sites). Lack of pericentromeric 1.688 also affected 1.688 expression in *trans* and was linked to the differential expression of genes involved in eggshell formation. We discuss our results with reference to the connections between POF, the 1.688 satellite and dosage compensation, and the role of the 1.688 satellite in hybrid lethality.

## 1. Introduction

Chromosome-wide targeting is widely appreciated to form part of dosage-compensation mechanisms, i.e., mechanisms that equalize the transcriptional output from, e.g., the single X-chromosome in males to the two X-chromosomes in females. Several different mechanisms have evolved that solve the gene dosage problem between the two sexes and have been described in the literature [1,2,3,4,5]. Importantly, although sex chromosomes often acquire chromosome-specific regulatory mechanisms, they are not an evolutionary dead end since examples of sex chromosomal reversion to autosomes do exist. One such model example is provided by the 4th chromosome in *D. melanogaster* (the Muller F-element). The 4th chromosome exhibits many indications of a relationship to the X-chromosome [1,2,6,7] and evolutionary studies suggest that the 4th chromosome was ancestrally an X-chromosome that has reverted to an autosome [8,9]. This reversion from a sex chromosome to an autosome may, in fact, explain the existence of two chromosome-wide regulatory systems in *Drosophila*.

In *Drosophila*, the most well-known example of a chromosome-wide system is the dosage compensation system which doubles the expression of genes on the male X-chromosome. The mechanism involves a combination of general buffering effects that act on all monosomic regions [10,11,12] and the specific targeting and stimulation of the male X-chromosome by the male-specific lethal (MSL) complex, which together result in an approximately 2-fold increase in gene expression [4,11]. The MSL complex consists of five proteins (MSL1, MSL2, MSL3, MLE, and MOF) and two redundant long non-coding RNAs (*roX1* and *roX2*) [4,13]. The *roX* RNAs are essential in maintaining the correct targeting of the MSL complex and the correct expression from the X-chromosome [14,15].

The second chromosome-wide regulatory system in *Drosophila* is represented by the protein Painting of fourth (POF) which is involved in the global regulation of the genes on *Drosophila’s* chromosome 4 [16,17,18,19]. The level of compensation mediated by POF is in the same range as the level of compensation mediated on the male X-chromosome by the MSL complex; furthermore the POF mediated compensation of the 4th chromosome is required for the survival of haplo-4 flies [17]. POF binding is restricted to chromosome 4-specific genes; however, binding necessitates the targeted genes to be under ‘heterochromatic pressure’ and so POF binding is dependent on heterochromatin protein 1 (HP1a) and the histone methyltransferase Setdb1 [17,20,21]. Taken together, these observations support a model in which POF originated as an ancestral dosage-compensation system with a stimulatory function and that this gene regulatory system was trapped on the 4th chromosome once the latter reverted from being an X-chromosome to an autosome with an accumulation of repeats and heterochromatin.

These genome-wide regulatory systems are remarkably dynamic and there are several examples of species in which the MSL complex machinery has evolved dosage compensation on new additions, e.g., neo X-chromosomes [22,23,24,25]. In most of the tested species within the *Drosophila* genus, POF specifically targets the Muller F-element both in males and females (corresponding to chromosome 4 in *D. melanogaster*) [1,2,26]. However, there are some species, e.g., *D. busckii*, in which POF targets only the male X-chromosome, and some other species, e.g., *D. ananassae*, in which POF targets the F-element both in males and females, as well as the male X-chromosome together with the MSL complex [26,27]. It remains unknown how the dynamics and acquisition of these chromosome-specific systems can be explained while they retain their high specificity. A growing amount of evidence suggests that repeated elements, such as from the expansion of microsatellites and transposable elements, are important in the de novo evolution of binding sites that can assist chromosome-specific targeting [24,25,28,29,30]. This argument has strengthened further evidence that in several *Drosophila* species both the X-chromosome and the F-element are over-populated by certain repetitive elements [31]. Forming de novo binding sites for chromosome-specific regulatory complexes seems to rely on the use of repeated elements and certain mutational pathways that depend on the genomic properties of a given species [24].

The 359 bp 1.688 g/cm^3^ satellite repeat is one of the four major satellites in *D. melanogaster* [7]. In contrast to the other three major satellites, the 1.688 is a sequence-wise complex satellite and is, remarkably, enriched ~50-fold on the X-chromosome compared to its presence on autosomes [32]. This extreme enrichment on the X-chromosome and the fact that it is barely detected on autosomes suggests a sex-specific role such as dosage compensation [30,31,33,34]. Evidence of a functional connection between the 1.688 satellite and dosage compensation has been lacking until recently when a functional link was demonstrated with the 1.688 satellite targeting both the MSL complex and POF [27,32,35,36].

In addition to the 1.688 repeats scattered on the euchromatic X-chromosome arm, the satellite is the principle component of the X-chromosome’s heterochromatin [37,38]. Interestingly, lncRNAs originating from the 1.688 satellite localises to centromeric regions of chromosomes; the depletion of these lncRNAs causes mitotic defects [39,40]. Furthermore, the deletion of the pericentromeric multi-mega base pair block of 1.688 repeats on the proximal X-chromosome was originally identified as the *Zygotic hybrid rescue* mutation (*Zhr^1^*). In a cross of *D. melanogaster Zhr^1^* females and *D. simulans* males, the otherwise lethal female hybrid offspring survive [41,42,43].

To further our understanding of the role of the 1.688 satellite DNA in chromosome-specific targeting and regulation, and to test if ancient dosage-compensation mechanisms have some remaining regulatory impact, we here provide an expression analysis of *Pof* mutant and 1.688 deletion mutants (*PoX2^Df1.688^* and *Zhr^1^*). We show that loss of *Pof* decreases not only chromosome 4 expression but also reduces the X-chromosome expression in males. The 1.688 deletion in *Zhr^1^* does not affect male dosage-compensation detectably; however, *Zhr^1^* in females causes a stimulatory effect on the X-linked genes with a strong binding affinity to the MSL complex (genes close to high-affinity sites).

## 2. Materials and Methods

### 2.1. Fly Strains

*Drosophila melanogaster* flies were cultivated and crossed at 25 °C in vials containing potato mash-yeast-agar. The mutant alleles analysed were *Pof^D119^*, *Zhr^1^*, and *PoX2^Df1.688^*. Oregon R was used as the wild type. The *Pof^D119^* allele is a deletion of around 1 kb uncovering the *Pof* coding region [17]. The *PoX2^Df1.688^* allele lacks the seven repeats of the 1.688 satellite downstream of *CG1840* [27]. The *Zhr^1^* allele lacks the ~5 Mbp pericentromeric 1.688 satellite block on the X-chromosome [41,42,43]. The *Zhr^1^* males used in the study carries a non-translocated wild type Y-chromosome. To avoid the risk of 1.688 satellite repeat expansion on the *Zhr^1^* X-chromosome, the genetic background of the mutants and the wild type were not isogenized.

### 2.2. Preparation of RNA Library and Sequencing

Adult males and virgin females from each strain were isolated during three fixed time periods after hatching to decrease potential age differences between samples: 6–8 h (three flies), 14–16 h (four flies), and 22–24 h (three flies). The adult flies isolated during each period were placed in a 1.5 mL RNAse-free Eppendorf tube and flash frozen in liquid nitrogen and 0.1 mL TRI Reagent (Ambion) per fly was immediately added to each tube. The samples were stored at −80 °C. Thus, four biological replicates were isolated for each of the four genotypes and two sexes, totaling 32 samples based on 10 individual flies (6–24 h) per sample. Total RNA was purified with a Direct-zol RNA MicroPrep kit. The extracted RNAs were quality controlled and quantified using a Fragment Analyzer instrument (Advanced Analytical) and the reagent (DNF-471-22-SS total RNA 15 nt). The RNA samples with an RNA integrity number >9 were chosen to make cDNA libraries. The libraries were generated using the NuGene system (Ovation RNA-Seq System 1–16 for *Drosophila*- PART NOS. 0350) and were fragmented (200 bp) with a Covaris E220 Focused Ultrasonicator prior to adapter ligation. The generated libraries were quality controlled and quantified using the Fragment Analyzer instrument and reagent (DNF-920-22-DNA 75–15,000 bp). Each library had an exclusive barcode sequence as a ligated adapter and the libraries were pooled to make multiplex libraries. The samples were sequenced on a HiSeq 2500 High Output mode (2 × 125 bp Paired-end, Illumina). Sequencing was performed by the SNP & SEQ Technology Platform in Uppsala. The RNA-seq data reported in this paper have been deposited in the Gene Expression Omnibus database (GSE136637).

### 2.3. Raw Data Processing

Adapter removal and quality trimming of raw FASTQ reads for all samples were done using Trimmomatic [44], version 0.36, with the following settings “ILLUMINACLIP: TruSeq3-PE.fa:2:30:10 LEADING:3 TRAILING:3 SLIDINGWINDOW:4:15 MINLEN:36”. Ribosomal RNAs were filtered using SortMeRNA [45], version 2.1b, (Bonsai Bioinformatics research group, University of Lille, France), using default settings and the rRNA databases provided. Trimmed and filtered reads were aligned to *Drosophila melanogaster* (*D. mel*.) reference genome, version 6.13, using the STAR. [46], version 2.5.4b, aligner with default settings.

### 2.4. Genome Read Counting

Read counts were obtained by FeatureCounts [47], version 1.6.3 (Department of Computing and Information Systems and Department of Mathematics and Statistics, The University of Melbourne, Australia). Gene read counts were quantified using the *D. mel*. 6.13 gene annotation file. The annotation file for repeats was computed using RepeatMasker [48,49], version 4.0.7 (Institute for Systems Biology, Seattle, WA, USA) with RepBase [50] libraries and settings ‘–nolow –species fly –gff’. The annotation file for 1.688 satellite sequence blocks was computed by using BLAST [51] and querying 1.688 sequences (Supplementary File 1) against the *D. mel*. 6.13 genome. BLAST hits were merged into blocks of 1.688 sequences by merging overlapping and adjacent (20 bp) alignments. A block was output to the annotation file (Supplementary File 2) if it was at least 269 bp long (~75% of 1.688 satellite sequence length). The depth of reads for each genomic position as a coverage track was visualised by Integrative Genomics Viewer (IGV), version 2.4.5 [52]. R, RStudio [53] (RStudio, Inc., Boston, MA, USA), and Python version 3.6 (The Python Software Foundation Beaverton, OR, USA) were used to process and analyse read counts.

### 2.5. Sample Similarity Heatmaps

Sample similarity was determined by subjecting read counts to a regularised logarithm transformation (RLT) [54], which produces variance stabilizing effects in the dataset, followed by hierarchical clustering based on Euclidian distance. Sample distances were plotted with the R heatplot.2 function from gplots [55].

### 2.6. Differential Expression

All differential expression calculations were made with DESeq2 [54] using mutant versus wild type comparisons. Gene expression analyses were done on the major *D. melanogaster* chromosomes 2L, 2R, 3L, 3R, 4, and X. Chromosome arms 2L, 2R, 3L, 3R were defined as autosomes (A). In the DESeq2 analysis, Wald statistics was applied to each gene by a negative binomial generalized linear model and the genes with Benjamini–Hochberg-adjusted *p*-values (*P*_adj_) < 0.05 were selected for further downstream analyses. The log_2_ fold change of significant differentially expressed genes (*P*_adj_ ≤ 0.05) per each chromosome were plotted by the ggplot2 R package [56]. The results of Wilcoxon signed-rank tests were added to the plots by ggsignif (https://github.com/const-ae/ggsignif) and ggpubr (https://github.com/kassambara/ggpubr) R packages to indicate significantly different chromosomes. The overlaps within the fly strains for the significant upregulated and downregulated genes were graphically visualised with the VennDiagram R package [57].

Expression of transposons were analysed in two ways: By quantifying read counts first to each transposon class, and then to each transposon locus in the genome. DESeq2 was used to create volcano plots of differentially expressed transposon classes. Dots were coloured in black if considered significant (*P*_adj_ ≤ 0.05 and up/downregulation log_2_ fold change ≥ 1), otherwise they were shown in grey. Labels are shown for black dots which also have up/downregulation log_2_ fold change ≥ 2. Labels were moved manually to avoid text overlaps. Redundant labels, such as for multiple variants of the same transposon, were removed. Overall differential expression of transposons per chromosome and locus was performed by importing DESeq2 output data into a Python script. The log_2_ fold values with *P*_adj_ > 0.05 were discarded. All entries which could not be assigned to one of the major chromosomes were assigned to ‘other’. Python library matplotlib [58] was used to create the box plots.

Differentially expressed 1.688 blocks were plotted in volcano plots with DESeq2. Entries on the X-chromosome were marked in red colour, otherwise blue. Grey dots are non-significant hits (*P*_adj_ > 0.05). Distribution of differentially expressed 1.688 satellite blocks on the X-chromosome was done by importing DESeq2 output data for each mutant versus wild type pairwise comparison into a Python script and plotted using matplotlib. The P_adj_ value from DESeq2 was used to classify blocks as either significant (≤0.05) or non-significant (>0.05), colouring them as black or grey, respectively.

### 2.7. Characterisation of 1.688 Transcripts between CG1840 and Sicily

Total RNA was isolated from five pairs of salivary glands of third instar females and males (Oregon R), three biological replicates per sex, with TRI Reagent (Ambion) according to the manufacturer’s protocol. The purified RNA was treated with RNAse-free DNase I (Thermo Fisher Scientific, Waltham, MA, USA, EN0525) and first strand cDNA was synthesised by RevertAid Reverse Transcriptase (Thermo Fisher Scientific, Inc., EP0441) using Oligo(dT)_18_, random hexamer, and gene-specific primers (sense and antisense), separately. The generated cDNA samples were analysed by PCR using Phusion Hot Start II DNA Polymerase (Thermo Fisher Scientific, Inc., F549L). The PCR primers used are listed in Table S1.

### 2.8. Functional Enrichment Analysis

The significant up- and downregulated genes for each fly strain versus wild type were separately introduced into FlyMine (http://www.flymine.org) for enrichment analyses following the Gene Ontology, Berkeley *Drosophila* Genome Project and Pathways protocol. Overlaps between the enriched lists created are provided in Tables S2 and S3.

## 3. Results

### 3.1. Pof^D119^, Zhr^1^, and PoX2^Df1.688^ Mutants Show Chromosome-Specific Differential Expression

**Figure 1:**
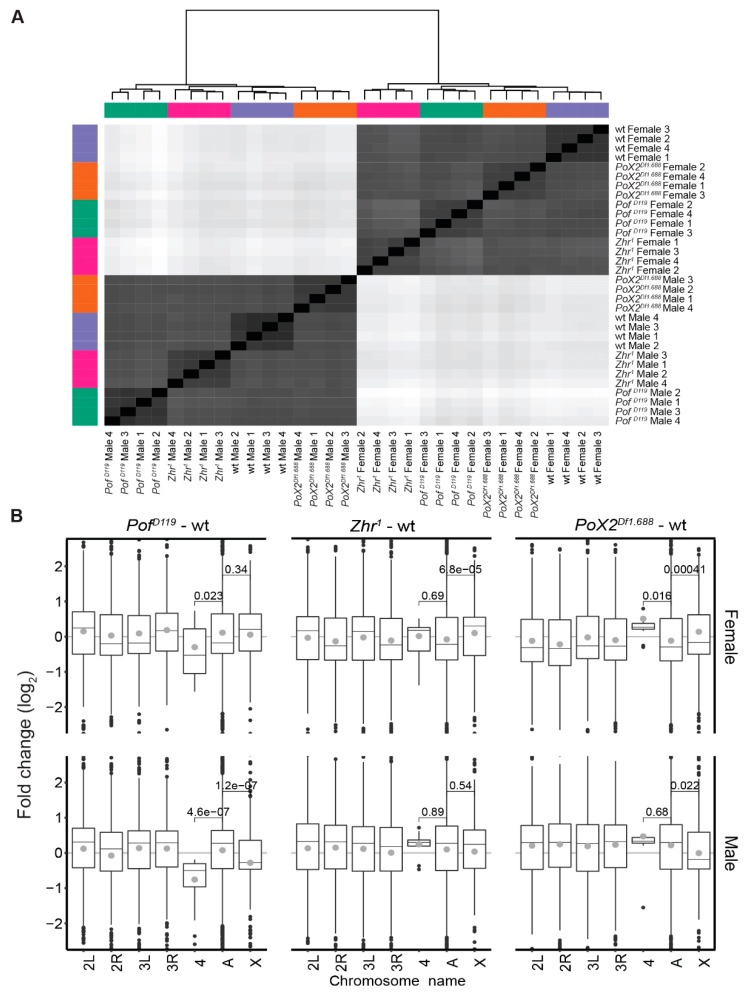
The gender-dependent genome differential analysis shows chromosome-specific alterations within genotypes versus wild type. (**A**) Samples were clustered and depicted in a heatmap based on Euclidian distances of gene read count RLT (regularised logarithm transformation). Sex is the major sample separator, followed by genotype. (**B**) Box plots representing expression ratios of whole transcripts (except satellites and transposable elements) for individual chromosome arms and chromosomes 2 and 3 combined (indicated A) in each mutant versus wild type. The box plots display median (line), average (dot), first and third quartiles (box), highest/lowest values within 1.5 × interquartile range (whiskers), and outliers. The statistical significance was determined by the Wilcoxon signed-rank test and p-values are indicated.

**Figure 2:**
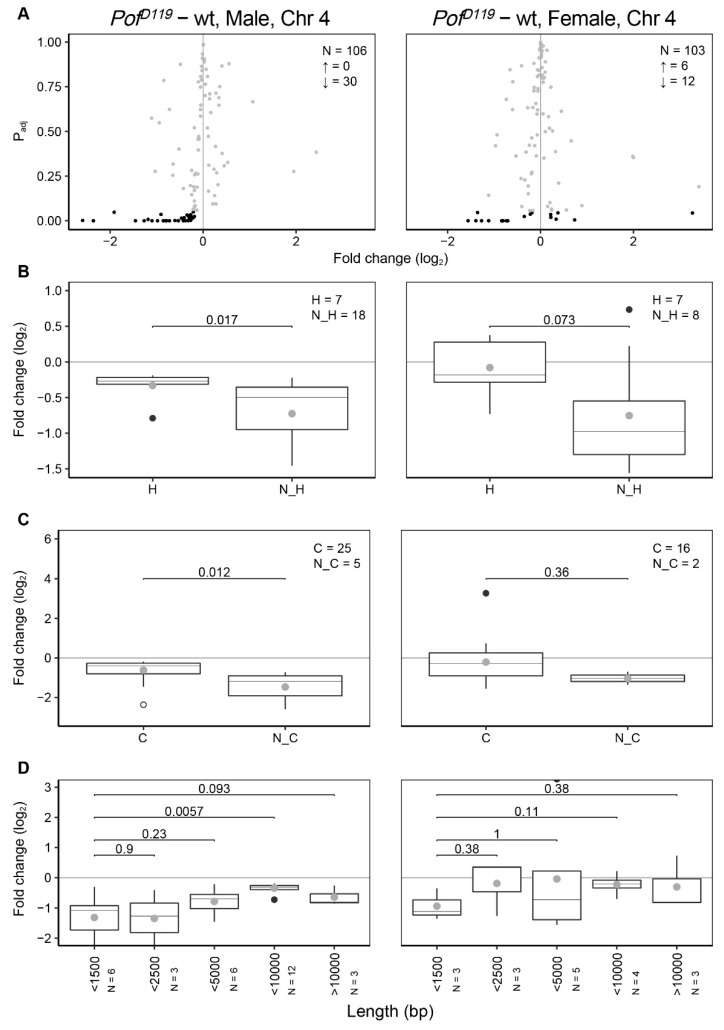
Feature analysis of significant differentially expressed genes on chromosome 4. (**A**) Differentially expressed genes on the 4th chromosome. N indicates the number of all altered genes, ↑ indicates the number of significant upregulated genes and ↓ indicates the number of significant downregulated genes. The grey dots refer to non-significant changes (*P*_adj_ > 0.05) and black dots indicate significant changes (*P*_adj_ < 0.05). (**B**) Comparison of expression ratios of housekeeping (H) and non-housekeeping (N_H) genes. (**C**) Comparison of expression ratios of coding (C) and non-coding genes (N_C). (**D**) Comparison of expression ratios of genes binned according to transcript length (bp) in which the binned transcript lengths represent the ranges 0–1500, 1500–2500, 2500–5000, 5000–10,000, and >10,000 bp, respectively. Only significant altered genes (*P*_adj_ < 0.05) were included in (**B**–**D**). The box plots display median (line), average (dot), first and third quartiles (box), highest/lowest values within 1.5 × interquartile range (whiskers), and outliers. The statistical significance was determined by the Wilcoxon signed-rank test and p-values are indicated.

**Figure 3:**
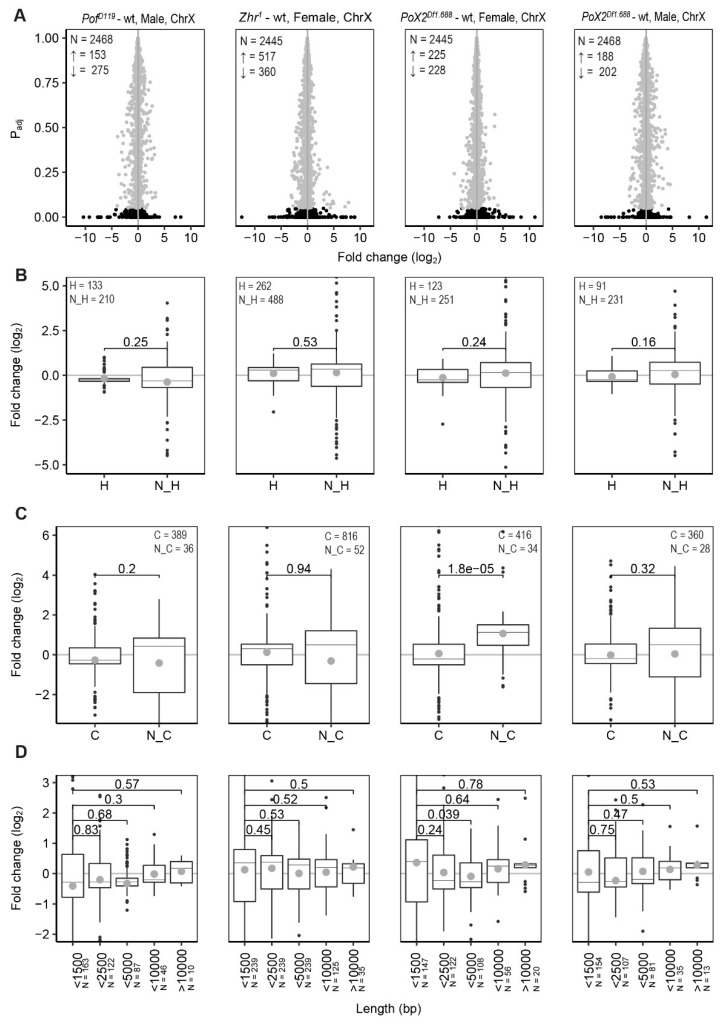
Feature analysis of significant differentially expressed genes on the X-chromosome. (**A**) Differentially expressed genes on the X-chromosome. N indicates the number of all genes, ↑ indicates the number of significant upregulated genes and ↓ indicates the number of significant downregulated genes. The grey dots refer to the non-significant changes (*P*_adj_ > 0.05) and black dots indicate significant changes (*P*_adj_ < 0.05). (**B**) Comparison of expression ratios of housekeeping (H) and non-housekeeping (N_H) genes. (**C**) Comparison of expression ratios of coding (C) and non-coding genes (N_C). (**D**) Comparison of expression ratios with genes binned according to transcript length (bp) in which the binned transcript lengths represent the ranges 0–1500, 1500–2500, 2500–5000, 5000–10,000, and >10,000 bp, respectively. Only significant altered genes (*P*_adj_ < 0.05) were included in (**B**–**D**). The box plots display median (line), average (dot), first and third quartiles (box), highest/lowest values within 1.5 × interquartile range (whiskers), and outliers. The statistical significance was determined by the Wilcoxon signed-rank test and p-values are indicated.

To investigate further the relationship between the 1.688 satellite element and chromosome-specific gene expression, we analysed and compared the genome-wide RNA expression in wild type and *Pof^D119^*, *Zhr^1^*, and *PoX2^Df1.688^* mutants. For this, we sequenced rRNA depleted RNA from adult males and females using an Illumina platform (four biological replicates per genotype). The calculated expression levels thus represent an average of different tissues and cell types. A heatmap of hierarchical clustering based on Euclidian distances produced from the RLT of the read count data showed that the samples were clustered perfectly by gender and genotype (Figure 1A). To find the main chromosome-specific differences between the genotypes, expression ratios for each chromosome were calculated both for the mutants and wild type (Figure 1B). We have previously shown that POF stimulates gene expression from the 4th chromosome [11,16,17,59] and as expected, in the *Pof^D119^* mutant, the gene expression on chromosome 4 was significantly decreased in both males and females compared with the other autosomes (Figure 1B, Figure 2A, and Supplementary Figure S1). Importantly, in *Pof^D119^* males, a significant decrease in average gene expression (7.68%) of the X-chromosome compared to autosomes was detected (Figure 1B). Of those genes classified as significantly differentially expressed (*P*_adj_ < 0.05), 275 were downregulated and 153 were upregulated (Figure 3A). Taken together, the results indicate an effect of the *Pof^D119^* mutant on male X-chromosome expression.

It has been suggested that the 1.688 satellite plays a role in chromosome-specific gene regulation, both in interactions with the dosage-compensating MSL complex [32,35,36] and as an optimal target for the chromosome 4 specific protein POF [21,27]. We were therefore interested in investigating any potential chromosome-wide effect on gene expression in the *Zhr^1^* mutant lacking the ~5 Mbp pericentromeric 1.688 satellite block [41,42,43]. A significant increase in the expression of X-linked genes was found in *Zhr^1^* females in which 517 out of 877 significantly altered genes on the X-chromosome were upregulated (Figure 1B, Figure 3A, and Supplementary Figure S2). Although it has been suggested that the enrichment of 1.688 satellite sequences on the X-chromosome stabilises the recruitment of the MSL complex [35,36], the average expression of the X-chromosome in males was not altered, in comparison with autosomes, upon deletion of the pericentromeric block of 1.688 satellite repeats (Figure 1B).

We have previously shown that the euchromatic *1.688^PoX2^* element promotes specific targeting of POF suggesting that this element retains a targeting function [27]. In wild type, POF exhibits female-specific targeting to a small number of X-chromosome sites—denoted as *PoX* sites [21]. This targeting is abolished in the *PoX2^Df1.688^* mutant females [27]. Therefore, we included the *PoX2^Df1.688^* mutant that lacks this specific 1.688 satellite repeat on the X-chromosome in our analysis. In *PoX2^Df1.688^* mutant females we observed a small but significant increase in gene expression, both on the X-chromosome and on the 4th chromosome, in comparison with autosomes (Figure 1B, Figure 3, and Supplementary, Figures S1 and S2). However, unlike the pericentromeric 1.688 satellite block, which has no detectable function in male X-chromosome dosage compensation, there was a small but significant reduction in expression of X-linked genes in male *PoX2^Df1.688^* (Figure 1B and Figure 3A). Notably, although the differences in X-expression observed in female and male *PoX2^Df1.688^* mutants are significant, these differences are not accompanied by more genes (in number) being up- or downregulated, respectively (Figure 3A).

We conclude that the loss of POF causes a significant decrease of gene expression on the 4th chromosome in males and females and, in addition, a significant decrease in X-chromosome expression in males. Removing the pericentromeric block of 1.688 satellites causes a significant increase of X-chromosome expression in females. Removing the specific short arrays of 1.688 repeats at the *PoX2* site causes a slight increase in the expression of the X-linked genes, as well as a small but significant increase in the expression of the 4th chromosome in females.

### 3.2. POF Stimulates Expression on the 4th Chromosome Preferentially on Short, Non-Coding, and Differentially Expressed Genes

We have previously delineated the relationship between POF and HP1a and the repressive role of HP1a on chromosome 4 genes [16,17,19,59]. We have shown that HP1a preferentially represses long and non-ubiquitously expressed genes along the 4th chromosome. We were therefore interested in studying the more detailed activities of POF as an HP1a concomitant on the 4th chromosome. Dividing the genes on the 4th chromosome into housekeeping and non-housekeeping genes confirms that loss of POF mainly affects non-housekeeping genes (Figure 2B) [11,59]. Next, we divided the genes on the 4th chromosome into either coding or non-coding and compared these two groups. Interestingly, in the absence of POF, the expression of non-coding genes in males shows a stronger reduction compared to coding genes (Figure 2C).

We have previously shown that a loss of HP1a affects gene expression differently depending on the gene length [59]. We therefore investigated whether differential expression of chromosome 4 genes in the *Pof^D119^* mutant correlates with transcript length. Short transcripts (<1500 bp) showed stronger reduction in expression compared to longer transcripts (Figure 2D). Taken together, our results confirm that POF stimulates the expression of chromosome 4 genes, and preferentially of differentially expressed genes. The effects of the *Pof^D119^* mutant on the 4th chromosome is similar in males and females. Short transcripts and non-coding genes show a more dramatic decrease in the male *Pof^D119^* mutant. Notably, these two classes are not mutually exclusive.

### 3.3. Relationship between X-Linked Transcriptional Alterations and MSL Complex Mediated Dosage-Compensation

Since loss of POF causes a stronger decrease of differentially expressed genes, short genes, and non-coding genes on the 4th chromosome, we also classified the X-chromosome genes according to these features and calculated the fold changes (Figure 3). Significant differences were observed in the size of effects when genes were classified as coding or non-coding, but the observed differences were small. We conclude that the small X-chromosome effects observed in these mutants (Figure 1B) are not clearly linked to some of these gene features.

**Figure 4:**
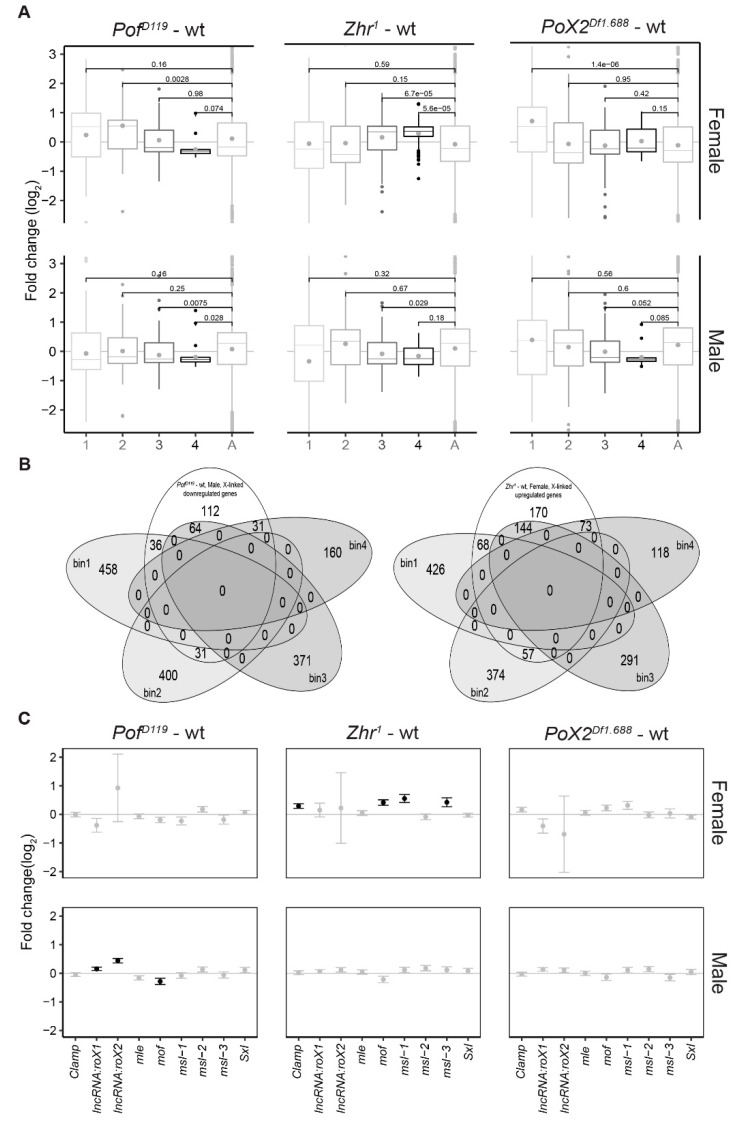
Relationship between X-chromosome specific effects and MSL (Male-Specific Lethal) binding levels. (**A**) Box plots representing average expression ratios of X-chromosomal genes binned based on their MSL binding strength (1 lowest to 4 highest). ‘A’ indicates the autosome chromosome arms combined (2L, 2R, 3L, and 3R). The MSL binding strengths from weakest to highest are shown by light grey to black, gradually. The box plots display median (line), average (dot), first and third quartiles (box), highest/lowest values within 1.5 × interquartile range (whiskers), and outliers. The statistical significance was determined by Wilcoxon signed-rank test and p-values are indicated. (**B**) Venn diagrams showing the number of downregulated genes on the X-chromosome in each MSL binding bin in *Pof^D119^*-wt males (left) and upregulated genes in *Zhr^1^*–wt females (right). (**C**) Expression analysis of the individual genes involved in the X-linked dosage compensation in the mutants versus wild type. The grey standard error bars refer to the non-significant changes (*P*_adj_ > 0.05) and black indicate significant changes (*P*_adj_ < 0.05).

Next, we asked whether relationships or correlations exist between our observed X-chromosome effects and the MSL complex dosage-compensation system. To characterise the significant X-chromosome effects observed in the mutants, all X-chromosome genes were divided into four bins based on their binding strength with the MSL complex [60,61]. Thus, bin 1 included unbound and weakly bound genes, while bin 4 included genes highly enriched in MSL protein bindings (Supplementary File 3). Genes on the autosomes (2L, 2R, 3L, and 3R) with very low enrichments for the MSL complex were considered as a control for the comparison (Figure 4A). In *Pof^D119^* females, we observed a descending order of gene expression ratios from bin 1 to bin 4. However, only the genes in bin 2 differed significantly from genes on the autosomes. Interestingly, in *Pof^D119^* males, the X-chromosome genes with stronger MSL complex binding (bins 3 and 4) were mainly responsible for the observed decrease in X-chromosome expression (Figure 1B and Figure 4A,B).

The increased expression of the female X-chromosome in *Zhr^1^* seems to be caused by an increased expression of those genes that in males show strong binding of the MSL complex, and thus have high expression in wild type (bins 3 and 4) (Figure 1B and Figure 4A,B). In *Zhr^1^* females the number of upregulated genes in bin 4 was higher than that of other genotypes versus wild type; these genes are also in closer proximity to the MSL high-affinity sites HAS and PionX (Supplementary Figure S3A,B).

In *PoX2^Df1.688^* females, we observed a significant up regulation of genes in bin 1. Notably, bin 1 consists of genes with a low expression level [15]. These results therefore suggest a de-repression of low expressed X-linked genes in *PoX2^Df1.688^* females. In *PoX2^Df1.688^* males, although there was a descending order of average expression ratios from bin 1 to bin 4; only the genes in bin 3 were significantly downregulated compared with the autosomes (Figure 4A).

Following the significant alterations of the genes close to high-affinity sites for the MSL complex in male *Pof^D119^* and female *Zhr^1^* mutants, we analysed the expression of some individual genes involved in X-linked dosage-compensation [4,13,62] in the mutants versus wild type (Figure 4C). The zinc finger protein CLAMP is a newly identified protein that cooperates with MSL2 in the binding of the MSL complex to high affinity sites [62,63,64,65]. Interestingly, although the expression of *roX1* and *roX2* was increased in *Pof^D119^* males, *mof* was significantly downregulated. Although speculative, this decrease may compromise the H4K16 acetylation and result in a decreased X-chromosome expression. In *Zhr^1^* females, *Clamp*, *mof*, *msl-1*, and *msl-3* were significantly overexpressed (Figure 4C). It remains to be tested if these observed increases in expression have a functional role in the increased X-chromosome expression observed in *Zhr^1^* females.

### 3.4. The Zhr^1^ Mutant Reduces the Expression of 1.688 Satellites in Trans

**Figure 5:**
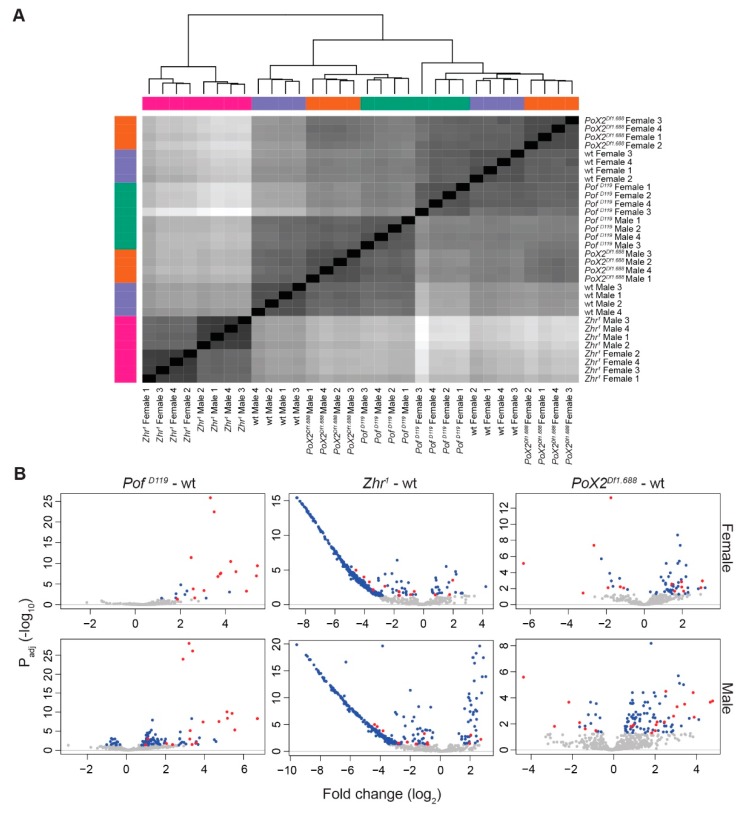
Satellites analysis in the mutants versus wild type. (**A**) Heatmap of Euclidian distances of RLT 1.688 satellite block read counts clusters the samples of *Zhr^1^* separated from *Pof^D119^* and *PoX2^Df1.688^*. (**B**) Satellite blocks on the X-chromosome are denoted in red, and satellite block on non-X-chromosomes are blue. Grey dots are non-significant hits (*P*_adj_ > 0.05). The largest difference of satellite block expression on autosomal and unclassified chromosomes is found in *Zhr^1^*.

In our distance analysis of gene expression profiles, the samples were robustly separated firstly by gender and secondly by genotype (Figure 1A). To test the RNA expression from 1.688 satellite repeats, we repeated the distance analysis but only included tests for expression of all blocks of 1.688 repeats throughout the genome. In this analysis, the *Zhr^1^* mutant genotype clearly outgroups. In fact, in the expression of 1.688 blocks, the *Zhr^1^* mutant outgroups from the other genotypes independent of sex, i.e., the differences between the *Zhr^1^* mutants and the other genotypes, were larger than the differences between males and females (Figure 5A). These results suggest that the lack of the pericentromeric 1.688 satellite repeats strongly affects the expression of the remaining 1.688 blocks as well (Figure 5A). To test this potential *trans*-effect of 1.688 expression, we analysed all separable 1.688 blocks individually (see Materials and Methods). The results showed that the lack of the pericentromeric 1.688 strongly reduces the expression of the remaining 1.688 blocks (Figure 5B). This reduction was mainly seen on 1.688 satellites that are not annotated on the X-chromosome (Figure 5B). In *Pof^D119^* females and males, we observe an increased expression of X-linked 1.688 satellites (Figure 5B). Our results thus confirm *cis*- and *trans*- activities of X-linked satellites and are consistent with other reports [39].

### 3.5. Expression Analyses of X-Linked 1.688 Satellites

**Figure 6:**
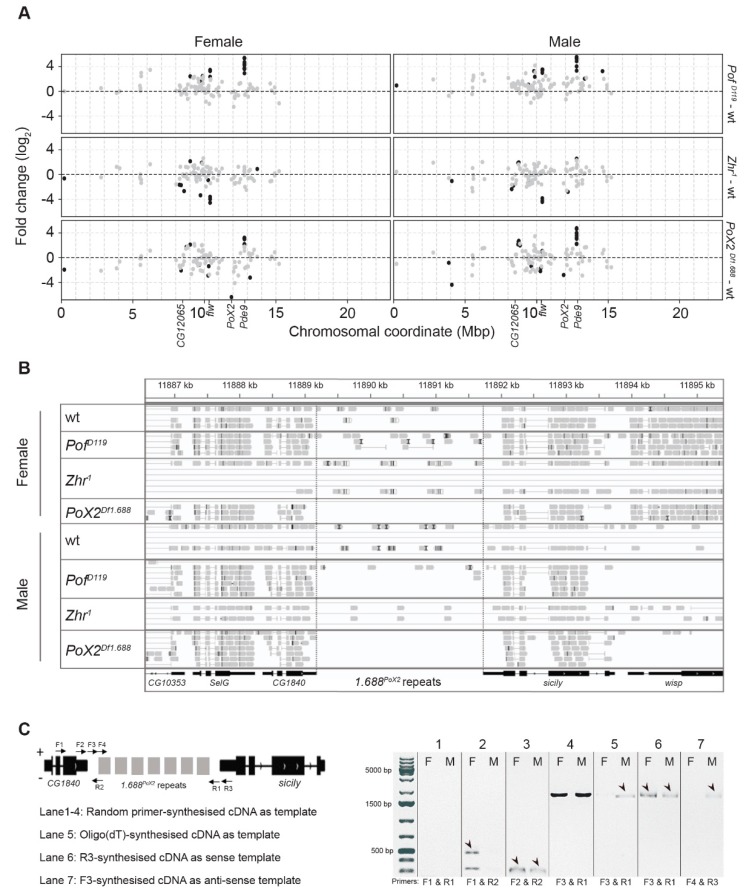
The 1.688 satellite expression analysis across the X-chromosome and at the *PoX2* locus. (**A**) Differential expression and X-chromosome distribution of 1.688 satellite blocks. Dots are coloured black if significant (*P*_adj_ ≤ 0.05) and grey otherwise. The *1.688^PoX2^* locus (indicated) is located at approximately 12 Mbp. (**B**) An Integrative Genomics Viewer screenshot with aligned reads at the *PoX2* locus in the different genotypes. Note that no reads map to the 1.688 region between *CG1840* and *sicily* in *PoX2^Df1.688^* mutant females and males. (**C**) Reverse transcription PCR using the indicated primer combinations confirm the short read-through and long non-coding RNA transcription from the 1.688 satellite at the *PoX2* site in wild type. In the gel electrophoresis panel, F and M indicate females and males, respectively.

As the 1.688 satellite blocks could be individually assigned, we specifically analysed the X-chromosome in which (in contrast to the autosomes) a large number of 1.688 blocks are distributed along the euchromatic arm [27,30,33]. In *Pof^D119^* females and males, we observed an increased expression of 1.688 satellite blocks on the X-chromosome; in particular a set of blocks located at coordinate 12.79 Mbp (which corresponds to 3.19 kbp of the fourth intron in the *Pde9* gene) (Figure 6A and Supplementary File 2). Notably, these blocks are also upregulated in *PoX2^Df1.688^* females and males. Note that *PoX2^Df1.688^* was generated by a CRISPR deleted 1.688 block at region 11.9 Mbp. A set of downregulated 1.688 blocks in the *Zhr^1^* mutant was concentrated at 10.39 Mbp (which corresponds to 1.01 kbp of the second intron in the *flw* gene) (Figure 6A). Another downregulated 1.688 block in *Zhr^1^* females corresponds to 767 bp of the second intron of *CG12065* at 8.5 Mbp. This 1.688 block is located 100 kbp downstream of the Chorion protein family genes at cytological band 7F1 (*Cp7Fa, Cp7Fb, Cp7Fc, Cp36,* and *Cp38*).

Next, we investigated whether we could specifically detect and identify RNAs generated from the *PoX2* locus. Since the 1.688 satellite is a multi-copy repeat with variations in sequence composition, we tested our stringency setting by mapping the reads from the different conditions to the genome assembly. We were encouraged to note that, in the sequence data from *PoX2^Df1.688^* mutants, no reads were mapped to the region downstream of *CG1840* (Figure 6B). This shows that the stringency setting we used can separate the *1.688^PoX2^* sequence variants from other 1.688 sequence variants. Therefore, the RNA-seq data confirm the existence of 1.688 transcripts that originate from the *PoX2* site both in wild type males and females (Figure 6B).

In our previous work to characterise *PoX2* [27], we detected a read-through transcript starting from the *CG1840* gene and progressing into the downstream 1.688 satellite repeat in wild type (i.e., the 1.688 repeat deleted in *PoX2^Df1.688^*). We therefore further surveyed the transcripts from the intergenic 1.688 satellite block in the *PoX2* downstream of *CG1840*. Different primer sets spanning *1.688^PoX2^* were used (Table S1) in reverse transcription PCR reactions in which total RNAs were extracted from the salivary gland cells of 3rd instar larvae; the transcripts were detected from the 1.688 satellite at the *PoX2* site (Figure 6C). The identified transcripts included a long non-coding RNA transcript (amplified with F3 and R1). We conclude that the 1.688 repeat downstream of *CG1840* is transcribed including a full-length read-through generating a 1.688 lncRNA. It has been reported that both strands of the 1.688 satellite DNA are transcribed in ovaries to provide a double-stranded RNA pool that may potentially lead to an RNAi-dependent regulation to maintain the silenced state of centromeric and pericentromeric 1.688 repeats [66]. Therefore, we tested whether the 1.688 repeat at *PoX2* is transcribed from both strands. Sense and anti-sense synthesised cDNAs were separately used as templates in reverse transcription PCR. The results showed that the *1.688^PoX2^* satellite region is only detected in sense direction in the salivary glands of wild type female larvae, while *1.688^PoX2^* transcripts were found in both directions in males (Figure 6C).

### 3.6. Transposon de-Repression in Zhr^1^ Mutant

**Figure 7:**
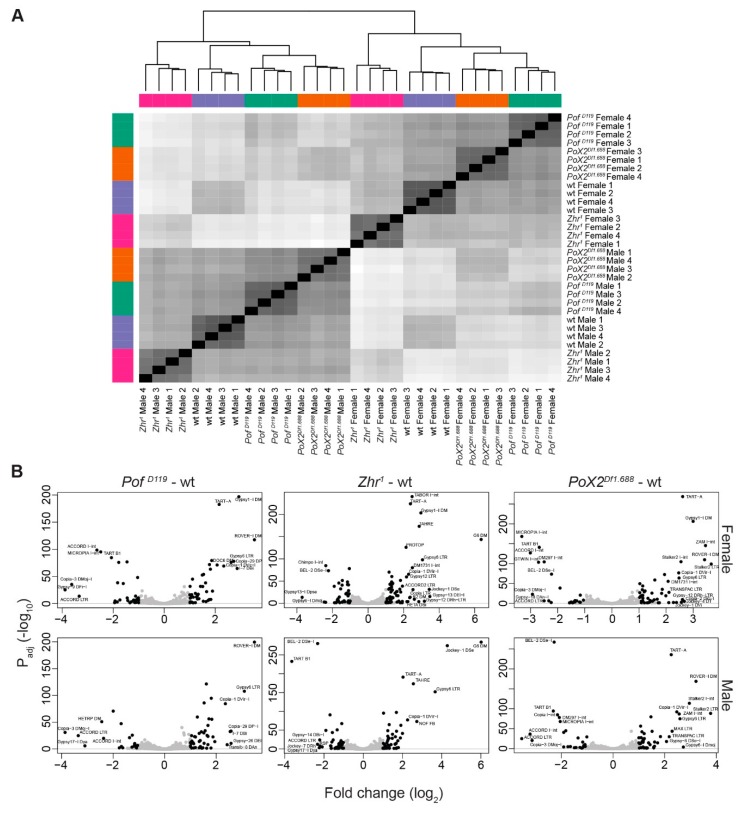
Genome-wide transposon analysis in mutants versus wild type. (**A**) Sample distance analysis based on RLT read counts of transposons cluster samples by sex and genotype. (**B**) Volcano plots of differentially expressed transposons. Transposons were predominantly de-repressed in *Zhr^1^* mutants, especially in females. Dots are coloured in black if significant (*P*_adj_ ≤ 0.05 and up/downregulation log_2_ fold change ≥ 1), otherwise grey. Labels are shown for black dots which also have up/downregulation log_2_ fold change ≥ 2. Labels were moved manually to avoid text overlaps and redundant labels were removed.

In several insect species, transposons consist of a constituent of satellite DNA [67]. Therefore, we analysed the differential expression of transposons when a multi-mega base pair satellite block was removed (as in *Zhr^1^*) or upon loss of its interacting regulatory systems (as in *Pof^D119^* and *PoX2^Df1.688^*). A sample distance analysis of RLT read counts of transposons (genome-wide) showed that the samples clustered primarily by gender and next by genotype (Figure 7A). In both females and males, *PoX2^Df1.688^* and *Pof^D119^* mutants clustered closely together in a further relationship with wild type, while *Zhr^1^* mutants were clustered over a greater distance (Figure 7A). We conclude that the mega base pair block deletion in *Zhr^1^* causes a stronger differential expression of transposon expression compared with *Pof^D119^* or *PoX2^Df1.688^* (Figure 7A). The identity and fold change of significantly differentially expressed transposons are shown in Figure 7B and the chromosomal locations are summarised in Supplementary Figure S4.

### 3.7. Female-Biased Genes Related to Eggshell Formation Show Increased Expression in Zhr^1^ Mutant Females

**Figure 8:**
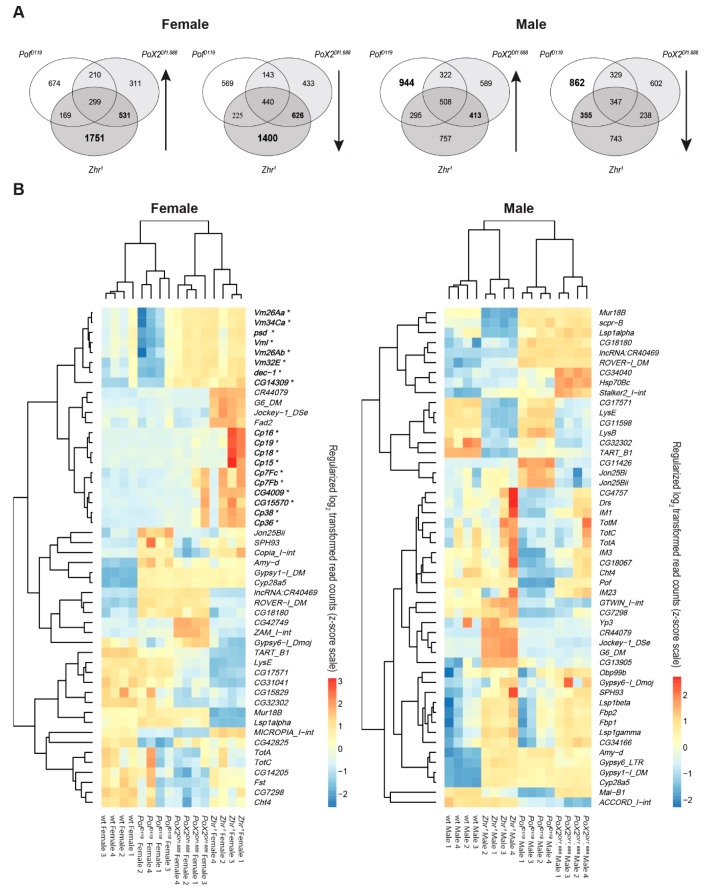
Co-regulated and most variable genes and transposons in the mutants versus wild type. (**A**) Venn diagram of co-regulated genes and transposons in the mutants versus wild type. (**B**) Heatmaps of the most variable genes and transposons in the genotypes (top 50) shows that several upregulated genes in *Zhr^1^* females are involved in eggshell formation (in bold, indicated by *).

We have previously hypothesized that the 1.688 satellite functioned in an ancient dosage compensation system involving POF targeting to the X-chromosome [27]. Therefore, in trying to find gene regulatory networks with genes and transposons that responded similarly in the different genotypes, we used Venn diagrams to compare all significant differentially up- and downregulated genes and transposons from each mutant. The highest numbers of differentially expressed genes in the mutants versus wild type were observed in female *Zhr^1^* and male *Pof^D119^*, respectively (Figure 8A). A high degree of overlapping upregulated/downregulated transcripts in females was found between *Zhr^1^* and *PoX2^Df1.688^* mutants (Figure 8A). In males, the highest number of co-upregulated transcripts was found between *Zhr^1^* and *PoX2^Df1.688^*, while *Pof^D119^* and *Zhr^1^* mutants showed the most common downregulated transcripts (Figure 8A).

A Gene Ontology enrichment analysis (excluding transposons) showed that most co-upregulated genes in *Zhr^1^* and *PoX2^Df1.688^* female mutants are involved in nucleic acid metabolic process and female gamete generation (Table S2), while the co-downregulated genes in these two mutants were mainly involved in developmental processes (Table S3). Notably, the gene families of Chorion protein and Vitelline membrane were found to have increased expression in *Zhr^1^* females and are among the top 50 most variable transcripts in our analysis (Figure 8B). The main variable transposons, e.g., *G6* and *Jockey-1* were also found in *Zhr^1^* in both females and males (Figure 8B).

We were intrigued by the increased expression in *Zhr^1^* females of a set of genes encoding Chorion proteins (*Cp16*, *Cp19*, *Cp18*, *Cp15*, *Cp7Fc*, *Cp7Fb*, *Cp38*, and *Cp36*), vitelline membrane proteins (*Vm26Aa*, *Vm34Ca*, *psd*, *Vml*, *Vm26Ab*, *Vm32E*), and other genes known to be involved in eggshell formation (*dec-1*, *CG14309*, *CG4009*, and *CG15570*) [68,69] (Figure 8B). The results suggest that the loss of the pericentromeric 1.688 satellite block in *Zhr^1^* affects eggshell formation.

## 4. Discussion

Chromosome targeting and regulatory mechanisms provide a good model to aid our understanding of the coordinated regulation of genes on an entire chromosome or even larger genomic regions. Chromosome-specific mechanisms are commonly recognised on sex-chromosomes as a means to restore the expression output between the heterogametic and homogametic sex. In fruit flies, two chromosome-wide systems have been characterised: The MSL complex dosage-compensation system that primarily acts on the male X-chromosome and, the system that we previously discovered, POF—the chromosome-specific regulation of genes located on the 4th chromosome, which is the first example of a chromosome-wide, autosome-specific gene regulatory system [16,17,18]. We have previously proposed that POF functioned in an ancient dosage-compensation system [2,26]. This hypothesis is supported by the later finding that the 4th chromosome was ancestrally an X-chromosome that reverted to an autosome [8,9] and our finding that in *D. ananassae* the POF protein is in close proximity to MSL3, i.e., POF is likely to be part of the MSL complex in *D. ananassae* [27]. Taken together, these findings support a hypothesis of POF having an ancient function in sex-chromosome dosage-compensation and that this function remains in some species.

How targeting of one specific chromosome evolves is still not understood; but repeated sequences in the form of satellites and transposable elements are considered to facilitate the evolution of chromosome-specific targeting [25,35,36]. We recently showed that a short array of 1.688 satellite repeats is essential to recruit the protein POF to a POF-high-affinity-site on the X-chromosome (*PoX2*), as well as to various transgenic constructs. Importantly, the 1.688 element has, for a long time, been considered in X-chromosome specific functions such as dosage compensation [30,31,33,34]. Experimental support for such a functional connection has been lacking until recently when the 1.688 satellite was functionally linked to both targeting of the MSL complex and targeting of POF [27,32,35,36]. Considering the rapid evolution and adaptation of regulatory mechanisms such as dosage compensation we hypothesised that POF and/or 1.688 may still show traces of dosage-compensation functions and we here tested this hypothesis using a transcriptome analysis. It is known that chromosome-wide regulatory systems act with different effect, the size of the effect depending on gene features such as housekeeping versus non-housekeeping genes, gene length, distance to high-affinity sites, and enrichment levels [11,15,59,61]. We therefore included these classifications in our analyses to increase the sensitivity.

### 4.1. Chromosome Specific Differential Expression in Male Pof^D119^ and Female Zhr^1^

As previously shown for other developmental stages, loss of *Pof* caused a significant reduction in expression output of genes from the 4th chromosome both in females and males. The decrease is more pronounced on differentially expressed genes as compared to housekeeping genes and also on non-coding genes as compared to coding genes. Note that these two criteria are not mutually exclusive. The results are consistent with the known stimulatory effect of POF on chromosome 4 [11,16,17,59]. In addition to the expected decrease on the 4th chromosome in *Pof^D119^* we observed a weak (8%) but significant reduction of X-chromosome expression in males. The observed reduction may be a remnant of an ancient function of POF in X-chromosome dosage-compensations. Interestingly, this reduction is without obvious phenotypic effects and we have recently suggested that tolerance to mis-expression is a common outcome in the evolution of sex-chromosomes [15]. The possibility that this is a remnant of a dosage-compensation function is supported by a more pronounced decrease of genes with high levels of MSL bindings, and genes located close to MSL high-affinity sites. Based on the current models of acquiring dosage-compensation [25,28] we assume that these genes acquire dosage-compensation early as an X-chromosome form. It has also been shown that full dosage-compensation is established earlier in the development of genes close to high-affinity sites [70].

Considering that the 1.688 satellite is ~50 times enriched on the X-chromosome compared to autosomes [30,31,32,34] it is tempting to assume a function (current or ancient) in dosage compensation. It has been shown that expressing siRNA from some specific variants of 1.688, increased MSL complex targeting and male viability in a genotype where both of these are compromised, i.e., *roX1* and *roX2* [35,36]. These results suggest an involvement of 1.688 satellites in dosage compensation. In the current study we tested if altered 1.688 content cause X-chromosome specific expression alterations using two genotypes: *Zhr^1^* with a deletion of almost the entire pericentromeric multi Mbp block of 1.688 elements; and *PoX2^Df1.688^*, which deletes an X-linked 1.688 block with remaining targeting functions of POF [27]. We did not observe any convincing decrease of male X-expression in these genotypes. However, in *Zhr^1^* females we observed a significant increase in X-chromosome expression. In support of this increase being a remnant of a dosage-compensation function, it is caused by an increase in the expression of genes with a high enrichment of the MSL complex in males. Why the loss of the pericentromeric 1.688 satellite block should lead to an increased expression in females, and whether that observation is connected to the increased expression of genes encoding proteins normally involved in male-specific dosage-compensation, remains to be tested. Notably, it also remains to be clarified if the reduction of X-chromosome expression in *Pof^D119^* males and the increased X-chromosome expression in *Zhr^1^* females are caused by a differential expression in most or all cell types or by a cell specific stronger effect.

### 4.2. Loss of the Pericentromeric 1.688 Satellite Region on the X-Chromosome Reduces Satellite Expression in Cis and Trans, and Induces Transposon de-Repression

A growing amount of evidence suggests that regulation, and in particular silencing, of HP1a enriched heterochromatin is important for proper development and co-ordinated gene expression. Nevertheless, flies are highly tolerant to dramatic changes in amounts of heterochromatic DNA. An example is the tolerance to loss of Y-chromosomes in males (X/0), as well as tolerance to additional Y-chromosomes both in males and females (X/Y/Y, X/X/Y, and X/Y/Y) [7]. The same is true for *Zhr^1^*. Although the X-chromosome in this mutant has lost >5 Mbp of pericentromeric 1.688 repeats, and thus a significant portion of the X-chromosome [7,71], it causes no obvious change in the phenotype. At the level of expression output, our results suggest that the 1.688 deletion in *Zhr^1^* causes a strong reduction of 1.688 expression in *cis*, as expected, but also in *trans*. The 1.688 sequence variants that show reduced expression in *Zhr^1^* are typically annotated to ‘unassigned chromosome’. We cannot exclude that some of these variants in fact originate from the *Zhr* repeated region, however, the results show that *Zhr^1^* does not induce de-repression of 1.688 variants in *trans*. In contrast to the reduced 1.688 expression in *trans*, we observed a de-repression of transposon expression in the *Zhr^1^* mutants. The elements *TART-A*, *G6*, *TAHRE,* and *Gypsy* were significantly de-repressed in both males and females.

In our previous study, we found that the removal of the 1.688 repeat at the *PoX2* locus, i.e., *PoX2^Df1.688^*, caused the loss of POF targeting also in *trans*, at a separate locus, *PoX1* [27]. In the current study, we identified 1.688 transcripts generated from the *PoX2* locus including a short read-through from *CG1840* and a separate long non-coding RNA transcript. Both sense and antisense transcripts from *1.688^PoX2^* in males were detected, which may indicate a double-stranded RNA pool that may potentially lead to siRNA formation and enhanced recruitment of the MSL complex to the chromosome; this would be in agreement with previously reported proposals [35].

### 4.3. The 1.688 Satellite Modulates Chorion Family Expression in Female D. melanogaster

Crosses between female *D. simulans* and male *D. melanogaster* are lethal to the female progeny and as such represent one of the very few exceptions to Haldane’s rule [72]. The lethality to hybrid daughters is mainly caused by incompatible pericentromeric loci comprising the *Zhr* 1.688 satellite repeats in *D. melanogaster*, which leads to failure of chromosome segregation during embryonic mitosis [41]. A possible mechanism is provided by the discovery of a long, non-coding RNA, produced from the *Zhr* locus, which has been shown to be localised at centromeric regions and depletion of which causes mitotic defects [39]. In our analysis of differentially expressed genes we found that those genes known to be involved in eggshell formation are upregulated in female *Zhr^1^*. This is of interest considering the significant differences in the ultrastructure of the chorion between *D. simulans* females and *D. melanogaster* males [73]. They showed that the chorion ridges in eggshells were thicker in *D. simulans* than in *D. melanogaster*. Considering the lack of the pericentromeric 1.688 satellite repeats on the X-chromosome, both in female *D. simulans* and in the *D. melanogaster Zhr^1^* mutant, we speculate that the *Zhr* locus directly or indirectly modulates genes involved in eggshell formation.

## Supplementary Materials

The following are available online at https://www.mdpi.com/2073-4409/9/2/323/s1, Supplementary Figure S1: Significant differentially expressed genes on the 4th chromosome in the mutants versus wild-type. Figure S2: Significant differentially expressed genes on the X-chromosome in mutants versus wild-type. Figure S3: Expression ratio analysis of significantly altered X-linked genes according to distance from HAS and pionX sites. Figure S4: Significant differential expression of transposons per chromosome in the mutants versus wild-type. Supplementary Table S1: PCR primers used in RT-PCR. Table S2: Gene Ontology enrichment analysis for up-regulated genes. Table S3: Gene Ontology enrichment analysis for down-regulated genes. Supplementary File 1: 1.688 satellite sequences used in BLAST. Supplementary File 2: Identified 1.688 sequence blocks. Supplementary File 3: X-chromosome genes divided into four bins based on their binding strength with the MSL complex.
