# Supplementary material for: Painting of Fourth and the X-Linked 1.688 Satellite in D. melanogaster Is Involved in Chromosome-Wide Gene Regulation
Source: Cells. 2020 Jan 30;9(2):323. doi: 10.3390/cells9020323 (PMC7072490; doi:10.3390/cells9020323)
Supplement: Supplementary file 1 [file cells-09-00323-s001.zip › Tousi_etal_Supplementary_Cells_r1.pdf]

Supplementary Material to

Painting of fourth and the X-linked 1.688 satellite in *D. melanogaster* is involved in chromosome-wide gene regulation

Samaneh Ekhteraei-Tousi, Jacob Lewerentz and Jan Larsson

Department of Molecular Biology, Umeå University, SE-90187 Umeå, Sweden

This PDF includes:

Supplementary Figure S1
Supplementary Figure S2
Supplementary Figure S3
Supplementary Figure S4
Supplementary Table S1
Supplementary Table S2
Supplementary Table S3

Legends for Supplementary Figures

Figure S1. Significant differentially expressed genes on the 4th chromosome in the mutants versus wild-type. Cytological location and fold change (log_2) of significantly altered genes on chromosome 4. The size of the dot indicates the mean of normalised counts of all samples.

Figure S2. Significant differentially expressed genes on the X-chromosome in mutants versus wild-type. Cytological location and fold change of significant altered genes on the X-chromosome (due to many significant altered genes with $P_{adj} < 0.05$, only genes with log_2 fold changes < -3 and > 3 are shown). Note that cytological region 7F shows several up-regulated genes including genes of the Chorion protein family (*Cp38*, *Cp36*, *Cp7Fc*, *Cp7Fa* and *Cp7Fb*) in *Zhr*^{1} and *PoX2*$^{Df1.688}$ females.

Figure S3. Expression ratio analysis of significantly altered X-linked genes according to distance from HAS and pionX sites. (*A*) Distance to HAS is shown for the genes within the different defined bins. (*B*) Distance to pionX is shown for the genes within the different bins.

Figure S4. Significant differential expression of transposons per chromosome in the mutants versus wild-type. All entries which were not assigned to one of the major chromosomes were assigned to 'other'.

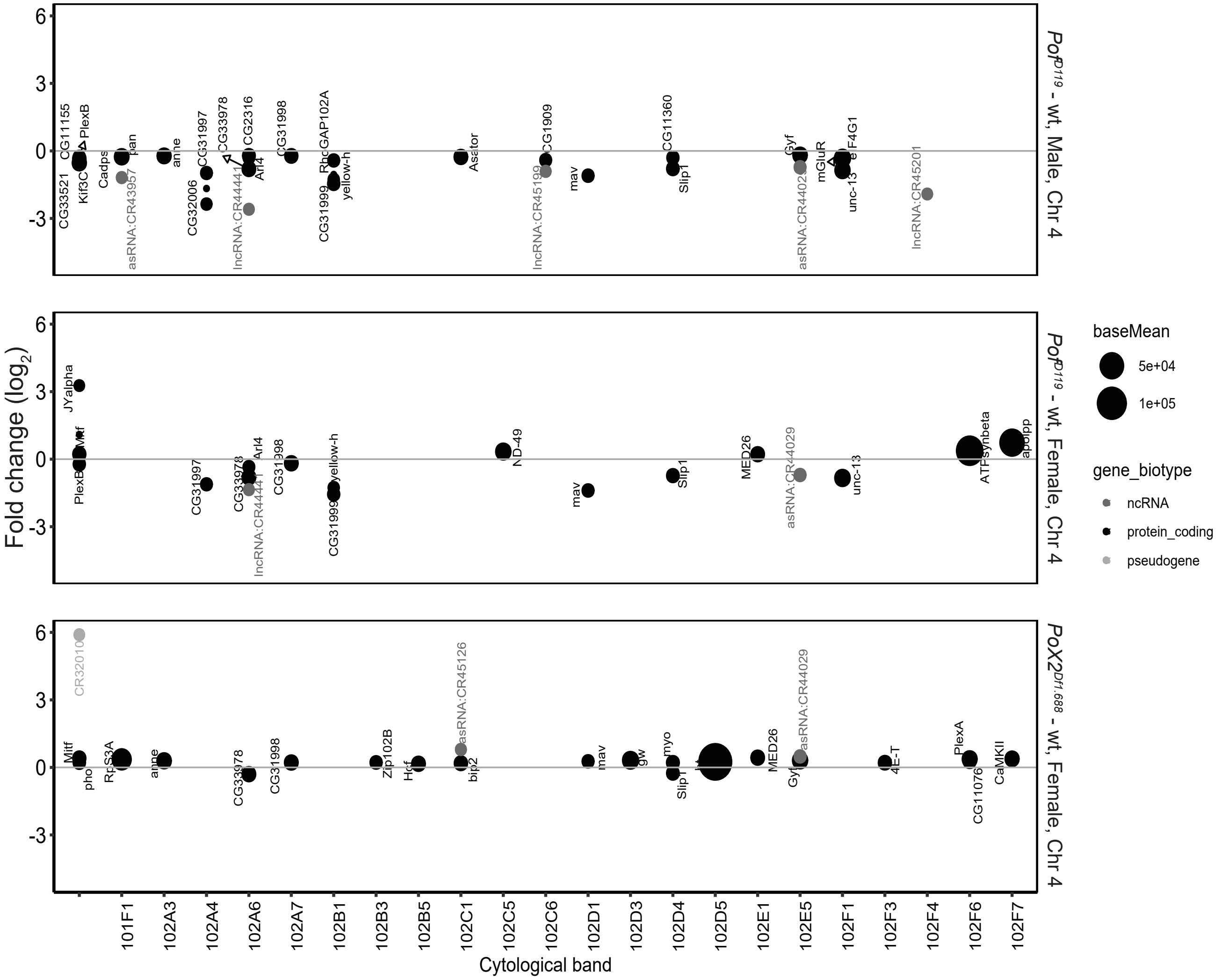

Fold change (log2)
Cytological band
PofD119 - wt, Male, Chr 4
PofD119 - wt, Female, Chr 4
PoX2Df1.688 - wt, Female, Chr 4
baseMean
5e+04
1e+05
gene_biotype
ncRNA
protein_coding
pseudogene
6
3
0
-3
101F1
102A3
102A4
102A6
102A7
102B1
102B3
102B5
102C1
102C5
102C6
102D1
102D3
102D4
102D5
102E1
102E5
102F1
102F3
102F4
102F6
102F7
CG33521
CG11155
PlexB
Kif3C
Cadps
asRNA:CR43957
pan
anne
CG32006
CG31997
CG33978
lncRNA:CR44441
CG2316
Arl4
CG31998
CG31999
RhoGAP102A
yellow-h
Asator
lncRNA:CR45199
CG1909
mav
CG11360
Slip1
asRNA:CR44029
Gyf
mGluR
unc-13
eIF4G1
lncRNA:CR45201
JYalpha
Mitf
PlexB
ND-49
MED26
ATPsynbeta
apolpp
CR32010
pho
RpS3A
Zip102B
Hcf
asRNA:CR45126
bip2
gw
myo
4E-T
PlexA
CG11076
CaMKII

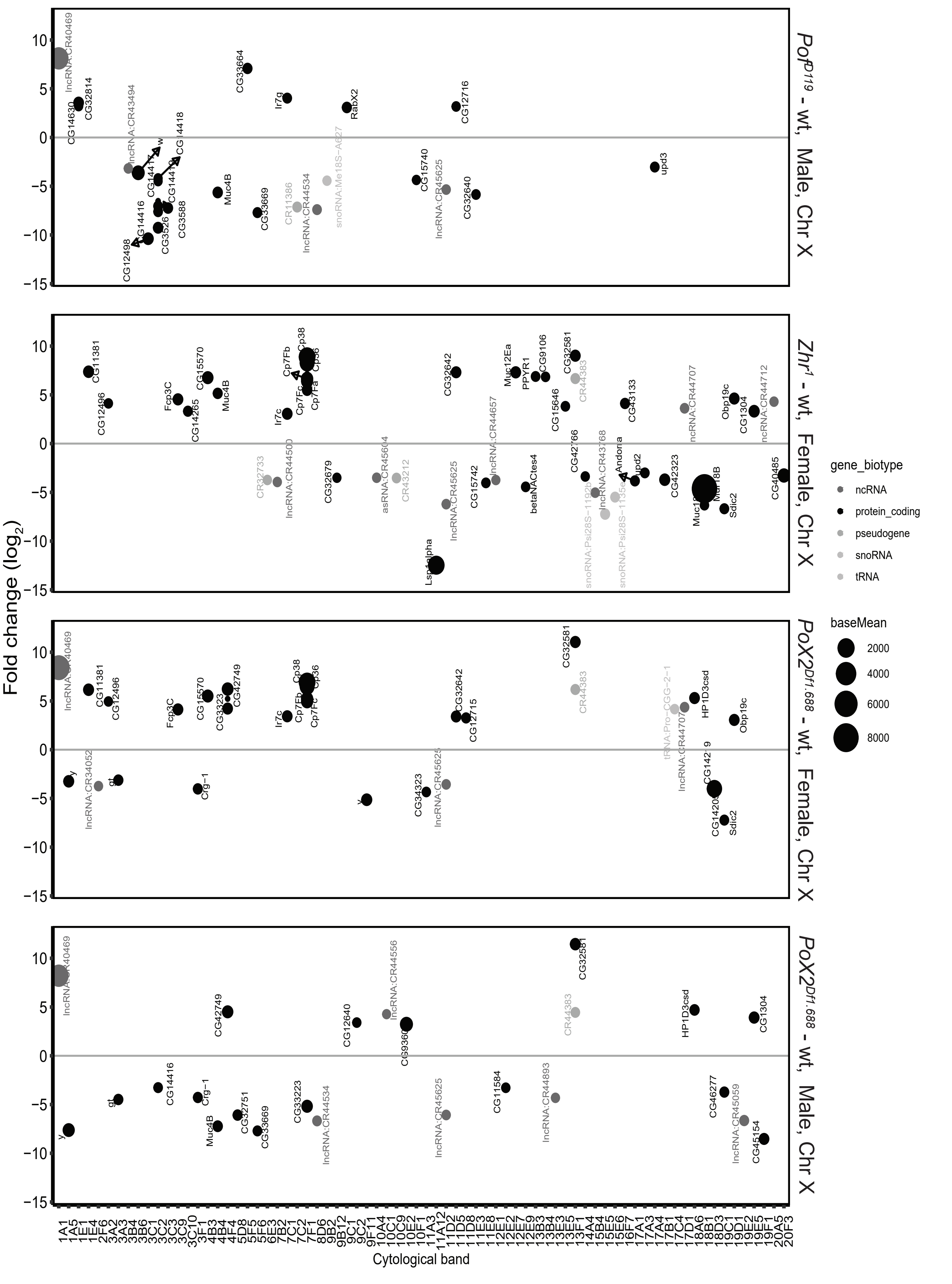

PoP^{119} - wt, Male, Chr X
Zhr^{1} - wt, Female, Chr X
$PoX2^{Df1.688}$ - wt, Female, Chr X
$PoX2^{Df1.688}$ - wt, Male, Chr X
Fold change (log_2)
Cytological band
gene_biotype
ncRNA
protein_coding
pseudogene
snoRNA
tRNA
baseMean
2000
4000
6000
8000

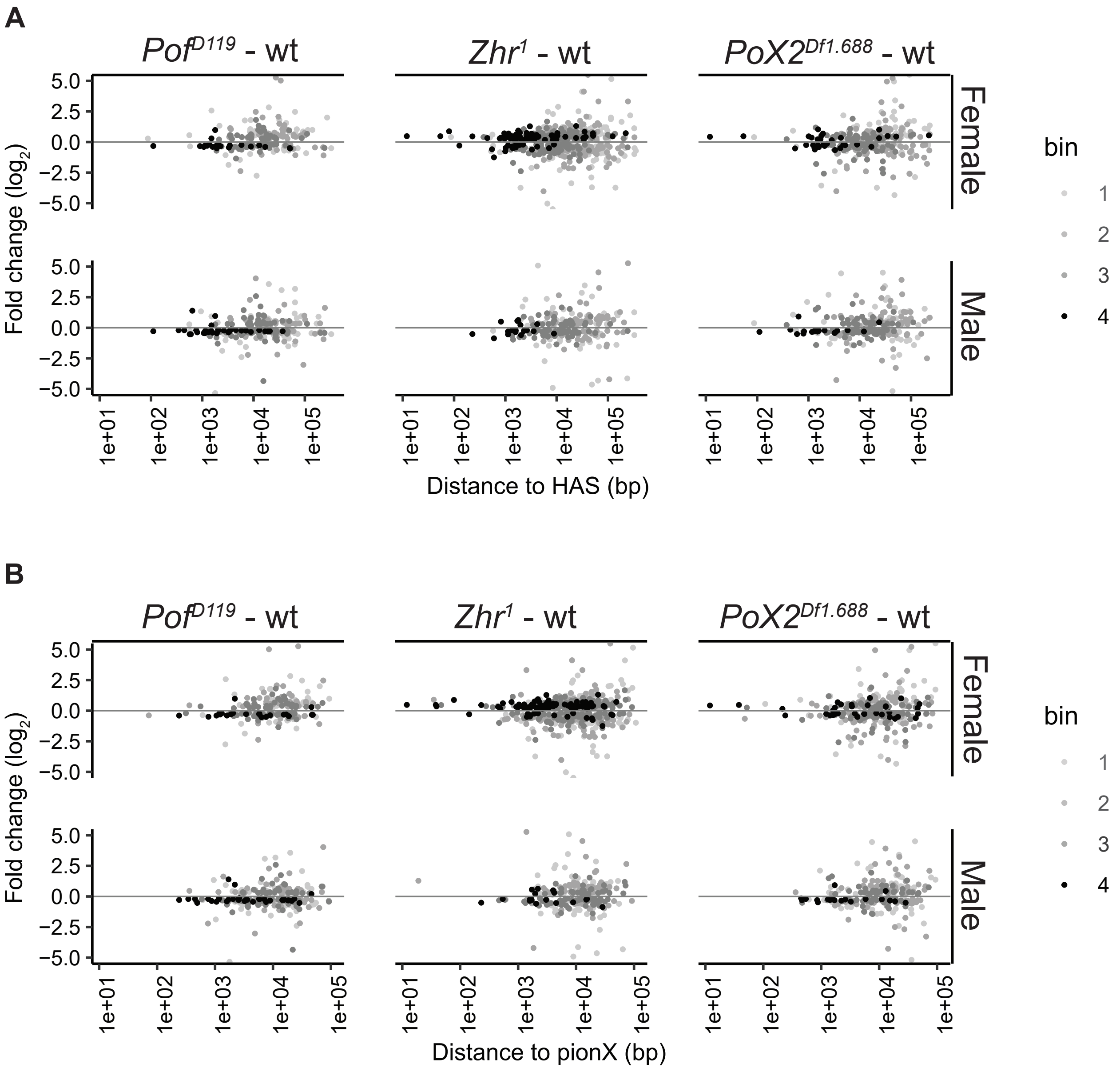

A
Pof^D119 - wt
Zhr^1 - wt
PoX2^Df1.688 - wt
Female
Male
Fold change (log2)
5.0
2.5
0.0
−2.5
−5.0
1e+01
1e+02
1e+03
1e+04
1e+05
Distance to HAS (bp)
bin
1
2
3
4
B
Pof^D119 - wt
Zhr^1 - wt
PoX2^Df1.688 - wt
Female
Male
Fold change (log2)
Distance to pionX (bp)

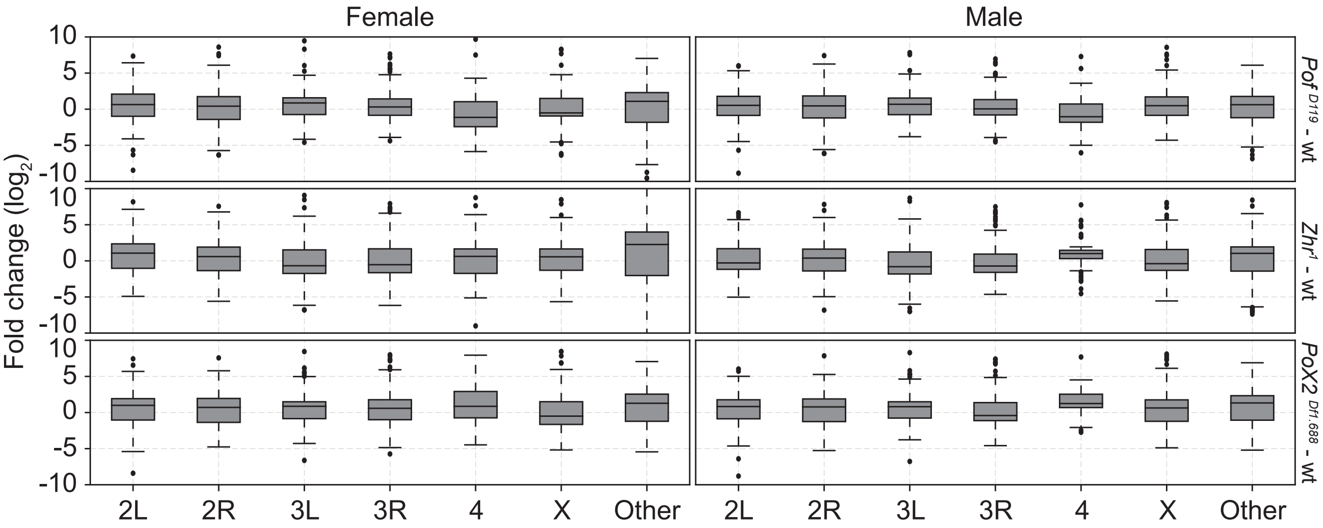

Female
Male
Fold change (log2)
10
5
0
-5
-10
2L
2R
3L
3R
4
X
Other
Pof D119 - wt
Zhr1 - wt
PoX2 Df1.688 - wt

Table S1. PCR primers used in RT-PCR

Primer	Sequence
F1	acgtccatgggacttgagac
F2	ccaactcacctctgtgatgc
F3	cagatgcttttgacttatttttgc
F4	caataattaaccacccctttcac
R1	gccatgcgtaatcttcttgac
R2	tgaaaagtgaaaggggtggt
R3	gcttttttgccatgcgtaatc

Table S2. Gene Ontology enrichment analysis for Up-regulated genes (mutants-vs-wt intersect)

Samples		Functional Enrichment analysis with flymine	P-value	GO number	Number of genes
Female	*Zhr*[1] & *PoX2*[Df1.688]	nucleic acid metabolic process	2.40093E-09	GO:0090304	187
		nucleobase-containing compound metabolic process	8.01502E-08	GO:0006139	199
		female gamete generation	1.15957E-07	GO:0007292	82
		oogenesis	5.19289E-07	GO:0048477	75
		heterocycle metabolic process	5.84051E-07	GO:0046483	201
		cellular aromatic compound metabolic process	1.8512E-06	GO:0006725	203
		organic cyclic compound metabolic process	4.82102E-06	GO:1901360	205
		RNA metabolic process	6.00943E-06	GO:0016070	161
		cellular nitrogen compound metabolic process	2.64798E-05	GO:0034641	244
		cellular process involved in reproduction in multicellular organism	4.47219E-05	GO:0022412	91
		germ cell development	4.91417E-05	GO:0007281	80
		negative regulation of cellular metabolic process	6.97975E-05	GO:0031324	73
		developmental process involved in reproduction	8.93772E-05	GO:0003006	87
		cell cycle	8.98213E-05	GO:0007049	80
		negative regulation of macromolecule biosynthetic process	0.000150518	GO:0010558	56
		negative regulation of cellular macromolecule biosynthetic process	0.000150518	GO:2000113	56
		negative regulation of biosynthetic process	0.000344787	GO:0009890	56
		negative regulation of cellular biosynthetic process	0.000344787	GO:0031327	56
		regulation of metabolic process	0.000566857	GO:0019222	168
		negative regulation of metabolic process	0.000629833	GO:0009892	84
		negative regulation of nitrogen compound metabolic process	0.000641373	GO:0051172	66
		gamete generation	0.000679427	GO:0007276	94
		regulation of macromolecule metabolic process	0.000897798	GO:0060255	158
		negative regulation of macromolecule metabolic process	0.001063682	GO:0010605	80
		chromosome organization	0.00109484	GO:0051276	74
		gene expression	0.00129007	GO:0010467	206
		regulation of chromosome organization	0.001620536	GO:0033044	27
		regulation of organelle organization	0.001658124	GO:0033043	48
		regulation of nitrogen compound metabolic process	0.002525102	GO:0051171	145
		cell cycle process	0.002666249	GO:0022402	63
		regulation of primary metabolic process	0.00349047	GO:0080090	146
		regulation of gene expression	0.005123208	GO:0010468	128
		regulation of cellular macromolecule biosynthetic process	0.005248183	GO:2000112	111
		regulation of cellular biosynthetic process	0.006405728	GO:0031326	114
		regulation of macromolecule biosynthetic process	0.006631289	GO:0010556	111
		regulation of biosynthetic process	0.006911928	GO:0009889	114
		regulation of cellular metabolic process	0.008870542	GO:0031323	150
		DNA metabolic process	0.009240153	GO:0006259	38
		regulation of mitotic cell cycle	0.009448323	GO:0007346	28
		RNA processing	0.010302842	GO:0006396	62
		chromatin organization	0.012607373	GO:0006325	54
		regulation of cell cycle	0.016402439	GO:0051726	38
		chromosome segregation	0.016435864	GO:0007059	29
		nuclear chromosome segregation	0.024194541	GO:0098813	27
		regulation of nucleobase-containing compound metabolic process	0.025805077	GO:0019219	107
		negative regulation of gene expression	0.030098203	GO:0010629	64
		negative regulation of nucleobase-containing compound metabolic process	0.039273205	GO:0045934	46
		negative regulation of RNA metabolic process	0.041201338	GO:0051253	45
		cellular component biogenesis	0.048034969	GO:0044085	115
		nuclear division	0.048316896	GO:0000280	38
	Zhr[1] & *Pof*[D119]	No enrichment found.			

	Pof^{D119}* & *PoX2$^{Df1.688}$	No enrichment found.			
**Male**	***Zhr^{1}* & *PoX2$^{Df1.688}$***	oxidation-reduction process	2.323120E-5	GO:0055114	65
		small molecule metabolic process	2.770730E-4	GO:0044281	77
	Zhr^{1}* & *Pof^{D119}	transmembrane transport	0.003487	GO:0055085	54
		peptidyl-histidine dephosphorylation	0.026500	GO:0035971	4
	Pof^{D119}* & *PoX2$^{Df1.688}$	transmembrane transport	0.011509	GO:0055085	53

Table S3. Gene Ontology enrichment analysis for Down-regulated genes (mutants-vs-wt intersect)

Samples		Functional Enrichment analysis with flymine	P-value	GO number	Number of genes
Female	*Zhr¹* & *PoX2^Df1.688*	regulation of biological quality	1.81181E-06	GO:0065008	156
		tube morphogenesis	3.05366E-06	GO:0035239	77
		anatomical structure morphogenesis	7.67735E-06	GO:0009653	173
		instar larval or pupal morphogenesis	9.71555E-06	GO:0048707	69
		post-embryonic animal morphogenesis	1.49707E-05	GO:0009886	70
		metamorphosis	2.25888E-05	GO:0007552	71
		tube development	3.60856E-05	GO:0035295	94
		animal organ morphogenesis	6.03118E-05	GO:0009887	98
		epithelial tube morphogenesis	8.96211E-05	GO:0060562	69
		tissue development	9.36618E-05	GO:0009888	126
		system development	0.000143005	GO:0048731	182
		localization	0.000160394	GO:0051179	230
		instar larval or pupal development	0.000186049	GO:0002165	76
		tight junction assembly	0.000265059	GO:0120192	15
		tight junction organization	0.000265059	GO:0120193	15
		animal organ development	0.000318012	GO:0048513	136
		post-embryonic development	0.000331423	GO:0009791	83
		epithelium development	0.000361483	GO:0060429	116
		regulation of supramolecular fiber organization	0.000509241	GO:1902903	23
		cell adhesion	0.000682887	GO:0007155	38
		imaginal disc morphogenesis	0.000809817	GO:0007560	59
		post-embryonic animal organ morphogenesis	0.000809817	GO:0048563	59
		actin filament-based process	0.00084479	GO:0030029	48
		biological adhesion	0.000885709	GO:0022610	38
		multicellular organism development	0.000951075	GO:0007275	234
		septate junction assembly	0.000973614	GO:0019991	14
		response to external stimulus	0.001324551	GO:0009605	118
		cell-cell junction organization	0.001723396	GO:0045216	18
		anatomical structure development	0.001741175	GO:0048856	260
		developmental process	0.001840576	GO:0032502	268
		neuron differentiation	0.002113269	GO:0030182	92
		actin cytoskeleton organization	0.002523564	GO:0030036	45
		cell morphogenesis	0.002605036	GO:0000902	74
		wing disc development	0.002782246	GO:0035220	56
		visual perception	0.002803827	GO:0007601	10
		tissue morphogenesis	0.002867023	GO:0048729	80
		cell junction assembly	0.002921145	GO:0034329	18
		cell-cell junction assembly	0.003213422	GO:0007043	17
		cell communication	0.004778284	GO:0007154	178
		fatty acid metabolic process	0.005055941	GO:0006631	26
		post-embryonic animal organ development	0.005178523	GO:0048569	63
		sensory perception of light stimulus	0.005951037	GO:0050953	11
		apical junction assembly	0.00620013	GO:0043297	15
		regulation of cytoskeleton organization	0.006954759	GO:0051493	29
		imaginal disc development	0.007730529	GO:0007444	72
		generation of neurons	0.008127213	GO:0048699	99
		cytoskeleton organization	0.008486211	GO:0007010	77
		monocarboxylic acid metabolic process	0.008487631	GO:0032787	33
		regulation of anatomical structure size	0.008569926	GO:0090066	40
		cellular developmental process	0.008980582	GO:0048869	175
		regulation of actin filament organization	0.009885038	GO:0110053	18
		neurogenesis	0.011524646	GO:0022008	102
		morphogenesis of an epithelium	0.012911463	GO:0002009	76
		actin filament organization	0.014594067	GO:0007015	28
		cell junction organization	0.015082952	GO:0034330	20
		fatty acid biosynthetic process	0.015522938	GO:0006633	18
		signaling	0.017339868	GO:0023052	172
		supramolecular fiber organization	0.018357274	GO:0097435	38
		stress-activated MAPK cascade	0.019117999	GO:0051403	19
		actin filament polymerization	0.022048841	GO:0030041	16
		regulation of protein polymerization	0.022048841	GO:0032271	16
		regulation of actin cytoskeleton organization	0.02466533	GO:0032956	21

	regulation of actin filament polymerization	0.025121699	GO:0030833	15
	transport	0.025822418	GO:0006810	173
	regulation of actin filament-based process	0.029451189	GO:0032970	21
	wing disc morphogenesis	0.030999209	GO:0007472	43
	cellular lipid metabolic process	0.031405219	GO:0044255	54
	actin polymerization or depolymerization	0.036640556	GO:0008154	17
	stress-activated protein kinase signaling cascade	0.03871083	GO:0031098	20
	response to wounding	0.043192662	GO:0009611	25
	establishment of localization	0.049487838	GO:0051234	176
	organic acid biosynthetic process	0.049787243	GO:0016053	25
	carboxylic acid biosynthetic process	0.049787243	GO:0046394	25
Zhr^{1}* & *Pof^{D119}	regulation of actin filament organization	2.44473E-07	GO:0110053	19
	cytoskeleton organization	5.08785E-07	GO:0007010	62
	regulation of supramolecular fiber organization	6.89475E-07	GO:1902903	21
	regulation of actin cytoskeleton organization	2.04118E-06	GO:0032956	21
	regulation of actin filament-based process	2.51268E-06	GO:0032970	21
	cell adhesion	4.92375E-06	GO:0007155	31
	biological adhesion	6.27439E-06	GO:0022610	31
	actin polymerization or depolymerization	1.4099E-05	GO:0008154	17
	actin filament organization	1.46891E-05	GO:0007015	25
	supramolecular fiber organization	2.92002E-05	GO:0097435	32
	regulation of biological quality	3.9206E-05	GO:0065008	99
	actin cytoskeleton organization	5.1667E-05	GO:0030036	35
	regulation of actin polymerization or depolymerization	6.00837E-05	GO:0008064	15
	regulation of actin filament length	6.00837E-05	GO:0030832	15
	regulation of anatomical structure size	9.05849E-05	GO:0090066	32
	regulation of protein polymerization	0.000100553	GO:0032271	15
	regulation of cytoskeleton organization	0.000108676	GO:0051493	24
	septate junction assembly	0.000145193	GO:0019991	12
	regulation of actin filament polymerization	0.000181459	GO:0030833	14
	cell-cell junction organization	0.000208767	GO:0045216	15
	actin filament-based process	0.000237588	GO:0030029	35
	tight junction assembly	0.00028379	GO:0120192	12
	tight junction organization	0.00028379	GO:0120193	12
	cell junction assembly	0.000331122	GO:0034329	15
	apical junction assembly	0.000418523	GO:0043297	13
	cell-cell junction assembly	0.000630948	GO:0007043	14
	actin filament polymerization	0.000795898	GO:0030041	14
	cell junction organization	0.003042418	GO:0034330	16
	regulation of protein complex assembly	0.004473027	GO:0043254	17
	protein polymerization	0.006840812	GO:0051258	16
	negative regulation of supramolecular fiber organization	0.007037348	GO:1902904	9
	chitin metabolic process	0.013000265	GO:0006030	18
	aminoglycan metabolic process	0.027772688	GO:0006022	20
	negative regulation of cytoskeleton organization	0.046234178	GO:0051494	9
	glucosamine-containing compound metabolic process	0.048449162	GO:1901071	18
Pof^{D119}* & *PoX2$^{Df1.688}$	regulation of actin cytoskeleton organization	5.19615E-08	GO:0032956	22
	regulation of actin filament-based process	6.50514E-08	GO:0032970	22
	regulation of actin filament organization	5.27581E-07	GO:0110053	18
	regulation of protein polymerization	3.28107E-06	GO:0032271	16
	actin cytoskeleton organization	5.20246E-06	GO:0030036	35
	regulation of actin filament polymerization	6.03006E-06	GO:0030833	15
	regulation of supramolecular fiber organization	7.94699E-06	GO:1902903	19
	actin filament organization	1.30172E-05	GO:0007015	24
	regulation of actin polymerization or depolymerization	1.84725E-05	GO:0008064	15
	regulation of actin filament length	1.84725E-05	GO:0030832	15
	regulation of cytoskeleton organization	1.9813E-05	GO:0051493	24
	cytoskeleton organization	2.26569E-05	GO:0007010	55
	actin filament-based process	2.54053E-05	GO:0030029	35
	actin filament polymerization	3.11151E-05	GO:0030041	15

		actin polymerization or depolymerization	3.19306E-05	GO:0008154	16
		septate junction assembly	5.5037E-05	GO:0019991	12
		protein polymerization	5.50452E-05	GO:0051258	18
		cell-cell junction organization	6.52539E-05	GO:0045216	15
		cell junction assembly	0.000104233	GO:0034329	15
		tight junction assembly	0.000108286	GO:0120192	12
		tight junction organization	0.000108286	GO:0120193	12
		cell junction organization	0.000145179	GO:0034330	17
		apical junction assembly	0.000150096	GO:0043297	13
		cell-cell junction assembly	0.000213381	GO:0007043	14
		regulation of biological quality	0.000643985	GO:0065008	89
		supramolecular fiber organization	0.000818836	GO:0097435	28
		regulation of protein complex assembly	0.001311718	GO:0043254	17
		negative regulation of supramolecular fiber organization	0.00339649	GO:1902904	9
		regulation of actin filament depolymerization	0.003796185	GO:0030834	5
		regulation of anatomical structure size	0.007275212	GO:0090066	27
		aminoglycan metabolic process	0.007304518	GO:0006022	20
		barbed-end actin filament capping	0.017133796	GO:0051016	4
		negative regulation of cytoskeleton organization	0.022741522	GO:0051494	9
		cell adhesion	0.038992092	GO:0007155	23
		biological adhesion	0.045826859	GO:0022610	23
	***Zhr*^{1} & *PoX2*$^{Df1.688}$**	No enrichment found.			
**Male**	***Zhr*^{1} & *Pof*^{D119}**	proteasome-mediated ubiquitin-dependent protein catabolic process	4.47179E-08	GO:0043161	35
		proteasomal protein catabolic process	2.47272E-07	GO:0010498	35
		modification-dependent protein catabolic process	5.30114E-06	GO:0019941	39
		proteolysis involved in cellular protein catabolic process	5.93297E-06	GO:0051603	41
		cellular protein catabolic process	9.31109E-06	GO:0044257	41
		modification-dependent macromolecule catabolic process	9.37154E-06	GO:0043632	39
		ubiquitin-dependent protein catabolic process	1.28599E-05	GO:0006511	38
		protein catabolic process	8.98436E-05	GO:0030163	41
		regulation of tube architecture, open tracheal system	0.000175295	GO:0035152	18
		regulation of tube size, open tracheal system	0.000630594	GO:0035151	13
		cellular macromolecule catabolic process	0.000674062	GO:0044265	46
		macromolecule catabolic process	0.000684153	GO:0009057	50
		regulation of tube size	0.001729021	GO:0035150	13
		respiratory system development	0.002897904	GO:0060541	29
		open tracheal system development	0.004354001	GO:0007424	28
		organonitrogen compound catabolic process	0.006052164	GO:1901565	47
		proteasomal ubiquitin-independent protein catabolic process	0.045025341	GO:0010499	8
	***Pof*^{D119} & *PoX2*$^{Df1.688}$**	No enrichment found.			
